# Effect of Intratrigonal Botulinum Toxin in Patients with Bladder Pain Syndrome/Interstitial Cystitis: A Long-Term, Single-Center Study in Real-Life Conditions

**DOI:** 10.3390/toxins14110775

**Published:** 2022-11-10

**Authors:** Pedro Abreu-Mendes, António Ferrão-Mendes, Francisco Botelho, Francisco Cruz, Rui Pinto

**Affiliations:** 1 Department of Urology, São João Universitary Hospital Center, 4200-319 Porto, Portugal; 2 Faculty of Medicine, University of Porto, 4099-002 Porto, Portugal; 3 i3S—Instituto de Investigação e Inovação em Saúde, Universidade do Porto, 4200-135 Porto, Portugal; 4 Life and Health Sciences Research Institute (ICVS), School of Medicine, University of Minho, 4710-057 Braga, Portugal

**Keywords:** botulinum toxin A, onabotulinum toxin A, bladder pain syndrome/interstitial cystitis, long-term treatment

## Abstract

The high percentage of treatment failures seen in patients with bladder pain syndrome/interstitial cystitis (BPS/IC) managed conservatively frequently demands invasive treatment options. We aimed to evaluate the long-term efficacy and adverse events of intratrigonal botulinum toxin injection in such circumstances, as well as to determine possible predictors of response to toxin treatment. A retrospective cohort study included 47 female BPS/IC patients treated with onabotulinum toxin A (OnabotA) in a tertiary hospital between the years 2009 and 2022. All patients received 100 U of OnabotA in ten injections limited to the trigonal area. Patients were divided into three groups based on their treatment response as responders, non-responders and lost to follow-up due to non-medical reasons. The clinical and surgical records of the individuals were retrieved, including the 10-point visual analogue scale (VAS), the number of treatments, the time between injections, and the age at the first injection. A total of 25 patients (>50% of the cohort) were long-term responders, but none of the evaluated parameters was a predictor for this circumstance: age, pain intensity, or duration of improvement following the injection. The time between injections was stable (around 1 year). No severe adverse events were registered. The intratrigonal injection of botulinum toxin in patients with BPS/IC was an effective and safe long-term treatment for patients’ refractory to conservative forms of treatment. Age, basal pain intensity, and time to injection request did not predict long-term response to OnaBotA.

## 1. Introduction

The European Society for the Study of Interstitial Cystitis (ESSIC) defines bladder pain syndrome/interstitial cystitis (BPS/IC) as a persistent or recurrent chronic pelvic pain, pressure or discomfort perceived to be related to the urinary bladder in the absence of any identifiable pathology which could explain this symptom [1]. It must be accompanied by at least one other urinary symptom, such as an urgent need to void or urinary frequency [1]. Therefore, BPS/IC diagnosis is still largely one of exclusion.

BPS/IC has no curative treatment as of yet [2]. Thus, symptom control, with the main focus on pain, represents a key part of BPS/IC management. The first line of treatment is centered on patient education and stress control to inform the patient about the uncertain evolution of the disease and to explain the importance of self-management by avoiding situations that may aggravate the symptoms [2]. When necessary, oral analgesic therapies and pharmacological agents intended to replenish the glycosaminoglycan layer and decrease urothelial permeability are used as the second-line treatment. When these measures are insufficient, surgical therapies may be introduced [3,4,5]. One option is the intratrigonal injection of onabotulinum toxin type A (OnaBotA) [6,7].

OnaBotA is a potent biological neurotoxin that accesses the neurons after binding the synaptic vesicle protein type 2C, a ubiquitous neuronal protein [6,8,9]. Once inside the neurons, the light chain of the toxin cleaves the proteins that are essential for the docking of the neuronal vesicles to the membrane in all types of neurons [9]. In sensory neurons, the trafficking of pain receptors from neuronal vesicles to the membrane of sensory neurons will be impaired and the release of CGRP and SP, two neuropeptides involved in neurogenic inflammation, will be substantially decreased [6,7,10]. In addition, a decrease of ATP release from urothelial cells occurs which may further impair bladder sensation [7,11,12]. The OnabotA-induced inhibition of the acetylcholine release from pre- and post-ganglionic parasympathetic neurons [13] is expected to have a limited role in BPS/IC, as this neurotransmitter is not involved in peripheral pain pathways and detrusor overactivity is a rare event associated with BPS/IC [9,14].

The application of OnabotA in the trigone is justified by the fact that this bladder region has the highest density of nociceptors [11,15,16]. Since 2004, multiple cohort studies and randomized placebo-controlled trials showed that OnaBotA was effective and safe in pain control in BPS/IC patients refractory to conservative treatment, in the dosage of 100 Units (U) [14,17,18,19,20]. In addition, trigonal injections of OnabotA also improve day and nighttime frequency, maximum functional bladder capacity, and overall quality of life, which are believed to result from less pain felt by patients during bladder filling [21]. 

One of the characteristics of OnabotA action is the limited duration of the effect, which, although variable, rarely extends for more than 12 months [7]. After this period of effect, BPS/IC symptoms tend to return, and repeated bladder injections are commonly requested by patients [21]. In previous prospective studies, overall, a statistically significant improvement in the pain as well as in other urinary symptoms has been reported, yet these results were mainly at short-term or after a few injection cycles [13,21,22,23]. Thus, information regarding the long-term effects and safety in real-life conditions is lacking.

We aim to report the results and safety of this intra-trigonal treatment in a real-life cohort of patients with a long-term follow-up. Differences between patients maintained in long-term therapy with intratrigonal OnaBotA treatment and those who stopped treatment, mainly for lack of response, were evaluated, looking for possible predictors of response. The rate of therapy maintenance was also assessed.

## 2. Results 

### 2.1. Patients and Demographics

A total of 47 BPS/IC female patients available in the hospital records and that were refractory to lifestyle changes and oral/intravesical therapies (including non-opioid analgesics and anti-depressant drugs and anti-histaminics) were included. All patients received at least one intratrigonal injection of 100 U of OnaBotA. A total of 193 procedures were performed as depicted in Table 1, varying between 1 (10 patients) and 14 (one patient). The proportion of patients that received four or more treatments was 48% (see Table 1).

The mean age of the patients at the time of the first injection was 50.7 (±14.5) years. The mean initial VAS score of the cohort was 5.7 (±1.7), and the mean follow-up is 8.8 (±4.2) years. The cohort demographics are presented in Table 2.

### 2.2. Duration of Effect per Injection

**Figure 1:**
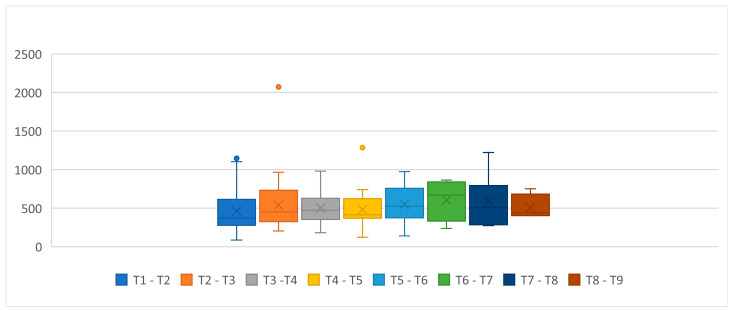
Median duration of effect between injections (*Y*-axis in days of effect duration counting time between an injection and the patient request for a new treatment).

The median interval between the injection and the patient’s request for a new injection is graphically shown in Figure 1, and it was 500.5 days (P25: 350; P75: 581), The duration of the effect of each treatment seems to be relatively stable during the follow-up. The median time between the first injection and the request for the second injection was the shortest with a median duration of 390 days (P25: 287; P75: 590). The median intervals for patients’ requests for another treatment increased afterwards, ranging between 414 days and 669 days. 

To investigate possible predictors of response, three groups of patients were defined. Group A comprised all patients currently in treatment, which included patients with the disease controlled confirmed at a visit or patients who already asked for a new injection). Group B comprised patients who were non-responders to OnaBotA (they could have shown treatment response at an initial phase of the OnabotA program but meanwhile the treatment lost efficacy). Group C included patients lost to follow-up due to non-therapeutic causes. 

When comparing the duration of effect between responders and non-responders, no differences were found, as shown in Table 3. We omitted group C in this comparison given the low number of patients and since the loss to follow-up was not related to the response to the toxin injections.

The 10th treatment onward medians were not represented in Figure 1 and Table 3 since only two patients reached that number of procedures.

Interestingly, of the nine patients that had a response duration below the 25th percentile after the first injection, only two abandoned the OnabotA program due to a lack of response. The other seven are still in treatment, having more than five injections. Moreover, these patients had in the following injections a duration of effect within the median time. This suggests that the duration of the effect of the first injection should not be used as a predictor of long-term treatment success or failure.

### 2.3. Global Treatment Maintenance

**Figure 2:**
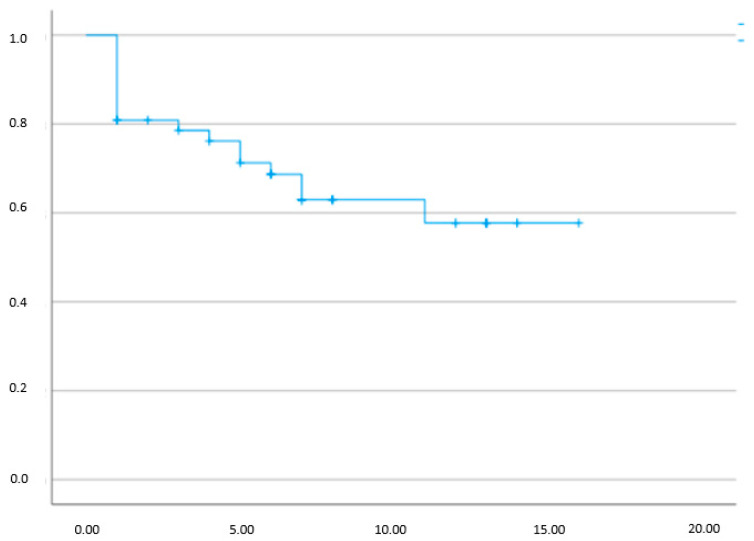
Kaplan-Meier curve of the cohort maintenance in OnaBotA treatment. The *X*-axis represents the number of injections, and the *Y*-axis represents the relative number of patients that were treated (100%). Each drop on the graphic line represents patients that lost treatment efficacy and the vertical dash along the graphic represents the moment patients were lost to follow-up. A total of 53% remained on treatment.

Given that we could analyze the total number of patients treated with OnaBotA and identify which of them are still in treatment per each treatment, we were able to organize a Kaplan-Meier treatment maintenance graphic. The results are shown in Figure 2. 

Notice that the graphic, which is not time-related, shows that more than half of the patients, 53%, had a favorable treatment response for a high number of treatments.

### 2.4. Possible Predictors

To evaluate the possible predictors, we compared the characteristics from group A and group B patients. The results are shown in Table 4.

As shown, 25 patients remain in treatment and 17 were non-responders to OnabotA. Notice that only one patient in group A had just one treatment (the patient has no symptoms after an injection carried out 15 months ago, with occasional flares easily managed with simple conservative measures).

The initial VAS score and the duration of the effect of the first treatment were not statistically different between responders (group A) and non-responders (Group B). The overall time between treatment and the subsequent request for reinjection was numerically inferior in the long-term responders in group A when compared to Group B, although the difference was not statistically significant (*p* = 0.72). 

### 2.5. Adverse Events

In terms of adverse events, and after analyzing all of the procedures, three types of complications were reported: lower urinary tract infection, straining during micturition, and acute urinary retention with a high post-voiding residual with the need to initiate clean intermittent catheterization (CIC). The results are presented in Table 5.

In 71 procedures, there was no specific mention of adverse events, and thus the adverse events were classified as omitted. Of those procedures with information available, 58 procedures had no adverse event, simple urinary tract infection UTI was recorded in 36 cases, and UTI with symptoms of straining was reported in 13 post-op procedures. Straining without UTI occurred after 14 procedures and straining with incomplete voiding, with the need for CIC, occurred in only one procedure (0.1%). No upper urinary tract infection was recorded.

## 3. Discussion

In the present paper, we describe our single-center experience with OnaBotA intratrigonal injection in the treatment of BPS/IC patients. To the best of our knowledge, this is the first long-term evaluation of a BPS/IC cohort with this treatment in real-life conditions. Previously, short and mid-term evaluations done in our and other departments had contributed to the validation of OnaBotA treatment in this disease [14,21].

The great majority, 37 patients, corresponding to 78% of the total cohort, requested more than one treatment. In recent years, we changed our approach and started to re-treat the patients that presented an unsatisfactory duration response to the first treatment at least one more time. This change was based on the perception that even patients with a poor response duration after the first injection could have a good response to posterior treatments. Such subjective opinion was now undoubtedly confirmed by the analysis of the present cohort.

There were no significant differences in age, the time to reinjection request, and pain intensity before the first intervention between responders and non-responders. Caution should be taken when interpreting the variables of time length for treatment requests after the first treatment and overall treatments, since group B includes nine patients that only underwent one treatment and, consequently, fewer patients contributed to this variable.

Contrariwise, patients that maintain a positive response to the OnaBotA treatment and longer time in treatment (5.4 ± 3.1 vs. 2.6 ± 2.1, *p* = 0.03), showed a longer period of follow-up in the urologic clinic (9.3 years vs. 3.4 years, *p* < 0.001). The reason is probably a selection bias because those that are more satisfied with the treatment results are more prone to continue in the program. 

Interestingly, the intervals between injections were longer than expected, compared to the duration reported in well-controlled cohorts or randomized clinical trials, in which only OnabotA treatment was allowed [5,21]. On average, the duration of each injection among the responders exceeded one year. This may indicate that OnabotA injections eventually combined with simple conservative measures and eventually oral medication with which patients had previous experience and could be used at their own decision (essentially non-opioid analgesic drugs, amitriptyline, the leukotriene receptor antagonist montelukast and antihistaminics [2,24]) may eventually substantially reduce the necessity of toxin injections, decreasing the burden that repeated injections cause to patients and health facilities. The median time between injection and a new request seems independent of the number of total injections and independent of the duration of the previous treatment, with the possible exception of the interval between the first injection and the request for a second treatment. 

The sustained duration of the effect, despite the increase in the number of procedures, suggests that intratrigonal sensory neurons do not develop tolerance to OnaBotA, even during long periods of administration. The use of botulinum toxin in other areas of bladder pathologies has shown the rare possibility of the development of antibodies as a cause of the appearance of resistance to the toxin [25,26]. It may be recalled that in a systematic review evaluating long-term treatment with intravesical OnaBotA in another urinary condition, overactive bladder, the data regarding the time between reinjections was heterogeneous among the analysed studies [27]. In some, the interval between injections was stable, while in others it could become either longer or shorter. 

As presented, seven of nine patients with a shorter time of effect after their first injection (lower than percentile 25) become good responders in subsequent treatments. As a matter of fact, they presented, in the following interventions, a time between injections and requests for retreatment similar to that found in the rest of the cohort. This could be justified by a cumulative therapeutic effect of the toxin, an adjustment on the parallel oral therapy, or by the stabilization of the disease. This finding could also be related to the inter-surgeon variation of the surgical technique and the process of reconstitution of OnaBotA. However, these two possibilities seem less probable, as if present they only occurred in the first treatment cycle.

Despite the significant number of patients with positive results that maintained the treatment in our cohort, approximately 53%, as seen in Figure 2, a large number of patients abandoned the long-term OnabotA program. One should remember that despite being effective in the long run, OnabotA injections can be rather unpleasant for BPS/IC patients. This had already been observed in overactive bladder and neurogenic detrusor overactivity cohorts, and reflects the low adherence to long-term reinjection programs [28]. Nevertheless, in our series almost half of the patients remained in the long-term program, exceeding the long-term adherence in OAB which may be as low as 10% [28]. Eventually, as the treatment for BPS/IC should be tailored-made for each patient, and since flares and remissions occur frequently, these different circumstances may introduce large variations in the time for patients to request another injection. [29]. 

The adverse effects observed in this group of patients are in line with other studies, with the risk of UTI and straining being the main concerns. This point should be fully discussed with the patients, since a UTI may markedly aggravate their symptomatology and the need for CIC will surely demand urethral manipulation. 

### Limitations

The main limitations of the study are the relatively small number of patients recruited, which were limited by the off-label nature of this treatment and the retrospective analysis of clinical records, and with the inherent risk of bias associated with the quality of the information in the files, including missing data. In addition, we could not provide reliable data on day and night time voiding frequency that we observed to decrease significantly in other studies [5]. However, from our perspective, it can be a more accurate representation of this treatment in real-world clinical practice. In this real-life scenario, the therapeutic effect of each treatment was determined by the time until a treatment request was made by the patients to avoid the bias of treatment delays due to fluctuations in the waiting list. 

The readers should also be aware that different from evaluating oral pharmacological treatment effects, the OnaBotA treatment is a surgical procedure and there could be inter-surgeon variations of the surgical technique. We believe that this bias has a low impact as the procedure is well standardized. 

Another limitation of this real-life study is relative to the use of other drugs other than OnaBotA by patients in this cohort. While patients could be medicated with a small number of pain-killers during the injections performed in a trial setting, these trial setting injections just represent a minor number of the treatments in the cohort. All of the other patients treated outside of a trial setting, and even trial patients once off the trial, were able to be medicated, or even automedicated, with non-opioid analgesics, anti-depressants and anti-histaminics to better control the symptomatology at their own discretion. Additional medication would probably be taken more often when the OnaBotA’s effect begun to decrease. However, this is a real-life setting, and BPS/IC patients rarely achieve symptom control with monotherapy (especially the patients represented in this cohort that are refractory to oral and intravesical treatments). Despite the fact that other medical therapies could have a role in explaining a more prolonged time of symptoms being under control and, consequently, a greater interval between OnaBotA injections, what is most important is that the BPS/IC condition was better controlled after OnaBotA treatments in responders.

The fact that this study reflects a real-life scenario could suggest that the mild adverse effects, being easily treated, could be underreported.

## 4. Conclusions

Intratrigonal injection of botulinum toxin A in patients with BPS/IC is an effective and safe long-term treatment option. Moreover, the duration of each treatment seems to be sustained along all of the treatments, even when the number of injections is high. Age, basal pain severity and short duration of the first treatment effect do not seem to predict whether a patient will be a long-term responder to OnaBotA. The main adverse effects are mild, simple UTIs and straining, occurring in a minority of procedures. A specific phenotype for OnaBotA responders is lacking, but given the widely positive effect of the treatment, it should be offered as a third line of therapy.

## 5. Material & Methods

This is a retrospective study, analyzing our cohort of patients diagnosed with BPS/IC and treated with intratrigonal botulinum toxin in a tertiary public university hospital in Porto (Porto, Portugal) between 2009 and 2022. The procedure and the OnaBotA preparation, in our institution, are carried out by different surgeons in the urology department and by different nurses in our surgical center.

BPS/IC is diagnosed in compliance with guidelines, following the exclusion of more common conditions through physical examination, patient history, uroculture, urinalysis, cystoscopy, urinary cytology, neurological examination and in some cases pelvic magnetic resonance. All patients performed bladder hydrodistension and urothelium biopsies, before initiating OnaBotA treatment, as part of the diagnostic workup. A total of 47 patients were identified.

The clinical and surgical records of the participants, available in our hospital database, were utilized for the analysis of data related to the interventions—every medical record such as written appointments, date of surgery and surgery details are available in the hospital database. The process of collecting the electronic records into files was conducted following the Ethics Committee’s approval for the study. Patients were assessed for pain intensity using a 10-point visual analogue scale (VAS) (results were from 0 to 10; a higher number corresponded to higher pain). For the evaluation of each patient, the authors accessed the number of treatments and the treatment duration (the duration between an injection and the request for a new one). The overall time of disease follow-up was also accessed. Adverse effects of the procedure, such as urinary tract infection, straining and urinary retention were analyzed when recorded.

Three groups of patients were defined to identify possible predictors of outcome. Group A comprised all patients currently in treatment (with the disease controlled after an injection or patients that already requested a reinjection); group B comprised patients who were non-responders to OnaBotA (they could have already shown treatment response but now are non-responders to OnaBotA and abandoned the treatment but not the clinic); and group C are patients lost to follow-up but for non-therapeutic reasons. 

A total of 25 patients met the criteria for group A, 17 met the group B criteria, and five met the criteria for group C. For the evaluation of possible predictors of long-term success, we compared the characteristics of group A with group B patients, such as age, the initial pain intensity evaluated by the VAS score, the overall treatment duration, and also the duration of the first treatment (excluding patients that only received one injection). Moreover, we evaluated whether a rapid loss of effect after the first treatment was predictive of a general treatment failure or the need for more frequent injections. The treatment maintenance was evaluated and displayed as a Kaplan-Meier graphic, providing information on the proportion of drop-outs and patients still in OnaBotA. The number of injections performed before abandoning OnaBotA treatment, as well as the number of injections performed by patients still in treatment, are represented in the graphic. 

Most treatments were performed outside of a trial, since from a total of 198 treatments, only 71 (35%) of them were in a clinical trial set EudraCT: 2014-001013-81, “Treatment of Bladder Pain Syndrome with Onabotulinum toxin A” (ProBaBle). This leads us to consider that these data reflect real-world practice.

### 5.1. Procedure Technique and Follow Up

**Figure 3:**
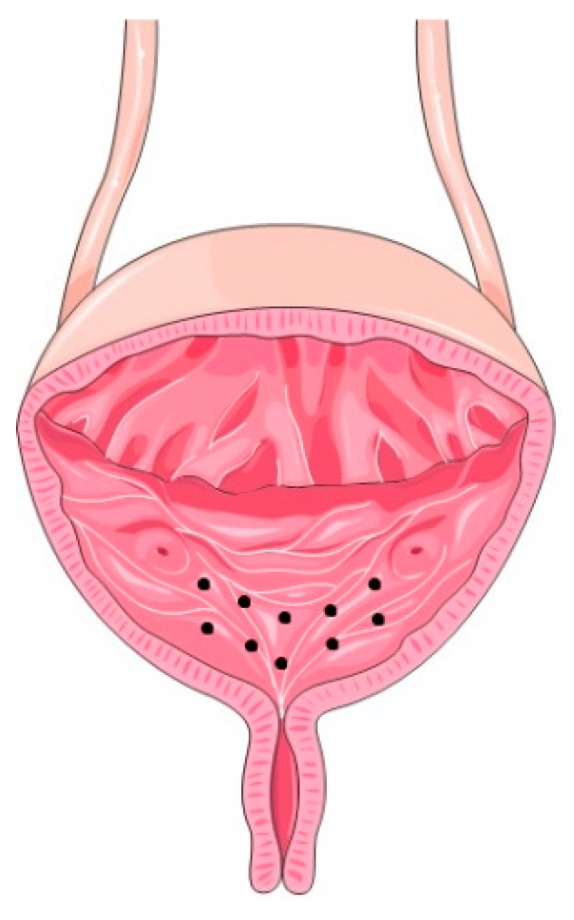
Schematic representation of the 10 bladder sites of OnaBotA injection.

In all our patients, the procedure, the drug and the dose administered were the same. The botulinum toxin A used was OnaBotA (Allergan, Irvine, CA, USA), and it was injected under light sedation through a 23-gauge needle (Coloplast A/S, Humlebaek, Denmark) inserted 3 mm into the trigonal wall with a 70°-lens cystoscopy control. A total of 100 Units were distributed throughout 10 sites (10 U per 1 mL saline)—Figure 3. A preoperative diagnostic workup was performed to guarantee that the individuals had a negative uriculture and no symptoms of cystitis. Patients were evaluated 2 to 3 weeks after the procedure to access early complications.

### 5.2. Statistical Analysis

The statistical program IBM^®^ SPSS^®^ v.28.0 (IBM Corp., Armonk, NY, USA) was used for data analysis. The Kolmogorov-Smirnov test was used to assess the normality of the distribution of continuous variables. Continuous variables with a normal distribution are presented as mean (±standard deviation). Non-normally distributed variables are reported as the median (percentile 25; percentile 75) and a Student’s t-test was used to compare the variables with normal distribution.

The authors chose to compare groups A and B for predictor analysis. Group C includes five patients that abandoned OnaBotA treatment early for reasons unrelated to the treatment or their clinical situation.

Clearance from the hospital Ethics Committee (Protocol number 337-21: Effect of intratrigonal botulinum toxin in patients with BPS/IC in a single center) was obtained.

## Figures and Tables

**Table 1:** Number of patients by the number of treatments.

Number of Treatments	N (Patients)	%
1	10	21.3
2	8	17.0
3	7	14.9
4	3	6.4
5	7	14.9
6	3	6.4
7	2	4.3
8	3	6.4
9	2	4.3
11	1	2.1
14	1	2.1
	47	100%

**Table 2:** Cohort demographics.

	Cohort
N (%)	47 (100%)
Age at first treatment	50.7 (±14.5)
Initial VAS	5.7 (±1.7)
Time between injection and request for another treatment (days)	500 (350–581)
Time between first injection and the request for the second treatment (days)	390 (287–590)
Number of Treatments	4.1 (±2.9)
Follow-up (years)	8.8 (±4.2)

**Table 3:** Median duration of the effect of each injection per injection, per group.

Treatment Response (in Days)	Group A	Group B	*p*-Value
After 1st treatment	447 (310–613)	337 (183–392)	0.57
After 2nd treatment	434 (308–553)	660 (327–1091)	0.64
After 3rd treatment	443 (343–622)	490 (279–673)	0.48
After 4th treatment	409 (377–647)	394 (184–571)	0.59
After 5th treatment	525 (405–845)	350 (140–562)	0.29
After 6th treatment	719 (399–848)		
After 7th treatment	543 (449–900)		
After 8th treatment	440 (403–681)		
After 9th treatment	450 (281–629)		

**Table 4:** Patients’ features as possible predictors, per group.

	Groups A and B	Group A	Group B	*p*-Value
N (%)	42 (100%)	25 (59%)	17 (41%)	
Age of 1st treatment	49.7 (±14.0)	51.8 (±11.5)	46.6 (±17.0)	0.29
Initial VAS	6.11 (±1.2)	5.9 (±1.3)	6.8 (±0.5)	0.27
Time between treatments	432.0 (305–645)	337.0 (182–747)	447.5 (310–613)	0.72
Time between 1st and 2nd treatment	350.0 (287–598)	447.5 (310–613)	337.0 (183–392)	0.94
Number of Treatments	4.3 (±3.0)	5.4 (±3.1)	2.6 (±2.1)	0.003
Follow-up (years)	6.9 (4.6)	9.4 (±3.9)	3.3 (±3.0)	0.001

**Table 5:** Total frequency of adverse events.

Adverse Events	Number	% (Total of 193)
Omitted	71	36.8%
None	58	30.1%
Urinary Tract Infection (UTI)	36	18.7%
Straining	14	7.2%
UTI + Straining	13	6.7%
Straining + incomplete voiding with the need for CIC	1	0.1%

## Data Availability

The data presented in this study are available on request from the corresponding author. The data are not publicly available due to Hospitals Ethics Committee policy.
